# Sporadic Medullary Thyroid Carcinoma with Paraneoplastic Cushing Syndrome

**DOI:** 10.1155/2019/6414921

**Published:** 2019-12-08

**Authors:** Aleksandra I. Pivovarova, Stephanie Patrick, Punuru J. Reddy

**Affiliations:** ^1^Alabama College of Osteopathic Medicine, 445 Health Sciences Blvd, Dothan, AL 36303, USA; ^2^University of Mississippi Medical Center, 2500 N State St., Jackson, MS 39216, USA; ^3^University of Tennessee Health Science Center, 910 Madison Ave, Memphis, TN 38163, USA; ^4^Decatur Morgan Hospital, Department of Internal Medicine, 1201 7th St. SE, Decatur, AL 35601, USA

## Abstract

Medullary thyroid cancer (MTC) is a rare form of neoplasm affecting the thyroid gland. This neuroendocrine tumor is capable of releasing active substances causing systemic manifestation in the form of flushing, diarrhea, and uncommonly, Ectopic Cushing's syndrome (ECS). MTC can be hereditary as a part of multiple endocrine neoplasm type 2 syndrome (MEN2) or arise sporadically. We report a case of a 74-year-old female presenting with chronic diarrhea, in whom diagnosis of sporadic MTC was delayed due to previous history of gastrointestinal (GI) disturbances. The patient developed liver metastases yielding ACTH dependent Cushing's Syndrome leading to abnormal clinical presentation and laboratory values driven by elevated cortisol level. Metastatic MTC should be considered in patients presenting with chronic diarrhea and weakness unexplained by other GI related causes.

## 1. Introduction

Medullary Thyroid Cancer is a neuroendocrine tumor of the C cells and accounts for 1–2% of thyroid cancers in the United States. While nearly all patients with familial MTC have *RET* germline mutation, only half of the sporadic MTC have somatic *RET* mutation present in tumor cells [1]. About 25% of MTCs are a part of familial MEN2 syndrome. Patients with MEN2 syndrome are inclined to develop MTC, pheochromocytoma, and hyperparathyroidism. In some MTC patients the disease can manifest systemically due to overproduction of calcitonin and other substances, such as catecholamines, serotonin, and histamine [2]. Majority of sporadic MTC patients will present with a palpable thyroid nodule, at which point approximately 75% of these patients would already develop cervical and 10% distant metastases [3]. The diagnosis is usually confirmed by a fine needle aspiration. After diagnosis of MTC has been established, measurement of serum calcitonin, carcinoembryonic antigen (CEA), genetic testing for germline *RET* mutations, and biochemical evaluation for coexisting tumors, especially pheochromocytoma should follow [1]. Measurement of calcitonin and CEA levels is used to determine a preoperative baseline and therapeutic response [4]. The first-line treatment for MTC is a surgical resection. Unfortunately, patients with metastatic MTC disease at diagnosis are rarely cured by surgery.

Only 0.7% of patients with MTC develops ECS, while MTC accounts for approximately 2.2–7.5% of patients with ectopic ACTH [5]. Ectopic Cushing's Syndrome from MTC is associated with significant morbidity and mortality, as secondary complications of hypercortisolism account for 50% of the mortality in MTC [6]. In the past, management of hypercortisolism was limited to decreasing metastatic tumor burden and using antiadrenal therapies such as ketoconazole, mitotane, and metyrapone [7]. Surgical intervention with bilateral adrenalectomy is another option available to some patients. However systemic therapy with tyrosine kinase inhibitors offers a further management strategy for disease control of metastatic and is now considered a first line therapy for ECS in the setting of unresectable disease or progressive, metastatic disease [8, 9].

## 2. Case Presentation

**Figure 1:**
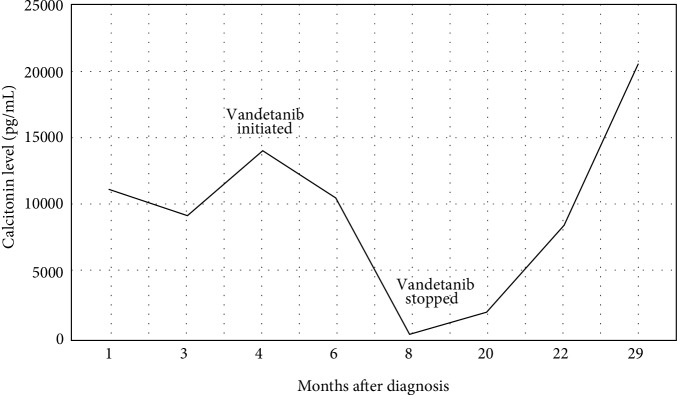
Calcitonin levels while on Vandetanib therapy. The graph depicts levels at baseline before the treatment was started, followed by initiation of Vandetanib, and subsequent rise in calcitonin levels after the therapy was stopped.

**Figure 2:**
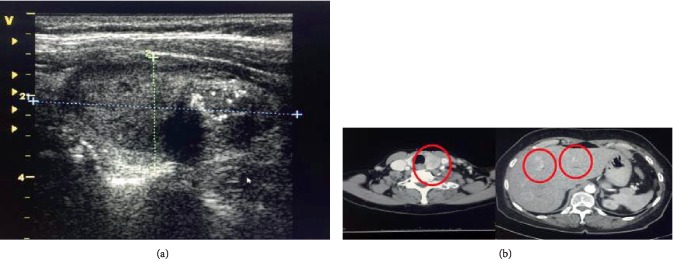
(a) Thyroid ultrasound demonstrating hypoechoic lesion. (b) Preoperative CT of the neck (left) and the abdomen (right) revealing cervical lymphadenopathy and liver metastasis.

**Figure 3:**
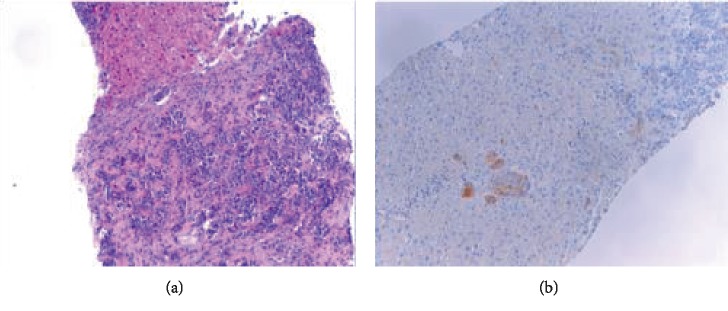
Liver biopsy showing metastatic medullary thyroid carcinoma cells (×10) (a) H&E stain. (b) Calcitonin stain.

A 74-year-old female presented with chronic diarrhea, weight loss, hematochezia, abdominal cramps, and fever. Her past medical history was significant for hypertension, metabolic syndrome, and perforated diverticulitis. Physical examination identified a nontender mass in her left submandibular area with no thyromegaly. The serological exam was remarkable for elevated chromogranin A level of 299 (normal <93). Suspicion for carcinoid tumor warranted an octreotide scan that identified increased uptake on the right side of the abdomen and left side of her neck. Laboratory tests revealed elevated calcitonin level, 11,290 pg/mL (ref. value <7.6 pg/mL) (Figure 1). Ultrasound of the neck identified a highly suspicious thyroid mass (Figure 2(a)), and a CT scan showed a heterogeneous thyroid nodule, and several enhanced lesions in the liver (Figure 2(b)). A liver biopsy revealed that tumor was of neuroendocrine origin consistent with the medullary carcinoma of the thyroid (Figure 3).

Patient had no family history of MTC or MEN syndrome. She underwent a total thyroidectomy followed by treatment with Vandetanib, which lead to resolution of diarrhea and normalization of bowel function. However, the drug was poorly tolerated, patient developed rash, fatigue, weakness, and nausea, and the dosage had to be tapered down and eventually stopped completely.

Two and a half years after the initial diagnosis of MTC, the patient returned to the clinic with edema, skin acne, elevated blood pressure, hyperglycemia, hypernatremia, and hypokalemia. Her symptoms were consistent with the Cushing syndrome. The Cortisol level was drastically elevated, 120.2 *µ*g/dL (ref. range for A.M. cortisol 6.2–19.4 *µ*g/dL). High dose dexamethasone suppression test was indicative of ectopic ACTH tumor. Brain MRI ruled out pituitary adenoma. It was concluded that liver lesions were the source of ectopic ACTH. To control hypokalemia, Aldactone was started along with a cortisol lowering ketoconazole, potassium, and calcium replacement. The patient was not a good surgical candidate, and bilateral adrenalectomy was not an option. While still in the hospital, the patient developed altered mental status and bleeding of colovaginal fistula. Her calcitonin level increased to 20,503 pg/mL (Figure 1). The patient's family decided in favor of comfort care, and less than two weeks later the patient passed away.

## 3. Discussion

Among the most common presenting signs of MTC are clinically detectable thyroid nodule, cervical lymph nodes, upper aerodigestive tract compression manifesting as hoarseness and/or dysphagia [1, 10]. Our patient's main presenting symptom was diarrhea, in which case MTC is not high on a differential. Initial presentation of diarrhea combined with the previous history of diverticulosis warranted a GI workup, which eventually lead to an accurate diagnosis of MTC. Unfortunately, diarrhea usually signifies advanced stage of MTC with metastatic disease [11].

While *RET* gene mutation is responsible for majority of familial MTC cases, 50% of sporadic MTC cases have somatic *RET* mutation in the tumor tissue [1]. With that being said, our patient did not possess a germline *RET* mutation, however genetic screening of patient's liver biopsy by NeoGenomics, performed after the patient passed away, demonstrated *RET *involvement by revealing a missense mutation.

Neuroendocrine tumors release calcitonin, calcitonin-gene related peptide, biogenic amines, and sometimes other active substances like ACTH, causing systemic symptoms. Ectopic ACTH secretion by a nonpituitary tumor leads to Cushing's syndrome, which is most commonly caused by corticotropin secreting pituitary tumor. Production of ACTH by MTC metastases is a rare occurrence and represents only about 5–10% of ECS [12]. Likewise, cancers such as small cell lung, thymic, pulmonary, pancreatic carcinoid tumours, and pheochromocytomas have the potential to produce ectopic ACTH [13]. The time between MTC diagnosis and ECS presentation can vary, with some declaring themselves even before MTC diagnosis, and others over 20 years after [14, 15]. In our case, ECS was detected almost three years after the initial diagnosis of MTC.

Once the type of cancer was identified, thyroidectomy and chemotherapy were quickly initiated. During the early stages of disease, the patient was treated with Vandetanib, the first drug approved in the United States for the treatment of progressive symptomatic MTC in patients with unresectable and/or metastatic tumors. Although the treatment with Vandetanib achieved resolution of diarrhea and return of normal bowel function, it had to be stopped due to poor tolerance. Even with the primary lesion being removed, liver metastases produced ectopic ACTH leading to Cushing syndrome, abnormal laboratory results, and overall worsening prognosis.

The Tyrosine kinase inhibitors Vandetanib and Cabozantinib have been approved by the Food and Drug Administration (FDA) for the treatment of progressive metastatic MTC [8]. Sorafenib, another kinase inhibitor, has been used off-label for treatment of metastatic MTC [16]. In a study by Barosso-Sousa, the treatment of metastatic MTC with ectopic ACTH syndrome with Sorafenib, reduced the cortisol and ACTH levels and lead to dramatic clinical improvement [17].

Vandetanib is considered a first-line therapy for ECS and should be used prior to bilateral adrenalectomy. Nonetheless, due to the patient's poor health and prior Vandetanib intolerance, she was not in the position to accept it and was started on comfort care instead. For patients with unresectable tumors and those who are unable to tolerate first line treatment, adrenal enzyme inhibitors such as ketoconazole, metyrapone, and etomidate can be used to control the symptoms along with glucocorticoid receptor blocker mifepristone. However, this is generally considered a suboptimal treatment [7]. Ultimately, late diagnosis due to an unusual presentation of MTC, and other existing comorbidities lead to poor outcome.
